# Wild canids and the ecological traps facing the climate change and deforestation in the Amazon Forest

**DOI:** 10.1002/ece3.10150

**Published:** 2023-06-09

**Authors:** Geovana Linhares de Oliveira, Arleu Barbosa Viana‐Junior, Paulo Henrique Santos Trindade, Iara Ramos dos Santos, Paula Cristina R. de Almeida‐Maués, Fernando Geraldo Carvalho, Daniel Paiva Silva, Øystein Wiig, Leonardo Sena, Ana Cristina Mendes‐Oliveira

**Affiliations:** ^1^ Laboratório de Ecologia e Zoologia de Vertebrados – LABEV, Instituto de Ciências Biológicas Universidade Federal do Pará Belém Pará Brazil; ^2^ Programa de Pós‐Graduação em Ecologia e Conservação da Universidade Estadual da Paraíba – UEPB Campina Grande – PB Brazil; ^3^ Centro de Estudos Avançados da Biodiversidade – CEABIO, Parque Tecnológico do Guamá Universidade Federal do Pará Belém Pará Brazil; ^4^ Unama Parque Shopping Belém Pará Brazil; ^5^ Laboratório de Ecologia e Conservação – LABECO, Instituto de Ciências Biológicas Universidade Federal do Pará Belém Pará Brazil; ^6^ COBIMA Lab, Departamento de Ciências Biológicas, Instituto Federal Goiano, Rodovia Geraldo Silva Nascimento Urutaí Goiás Brazil; ^7^ Natural History Museum University of Oslo Oslo Norway

**Keywords:** *Atelocynus microtis* (short‐eared dog), *Cerdocyon thous* (crab‐eating fox), ecological traps, general linear model (GLM), niche modeling, *Speothos venaticus* (bush dog)

## Abstract

Ecological traps occur when species choose to settle in lower‐quality habitats, even if this reduces their survival or productivity. This happens in situations of drastic environmental changes, resulting from anthropogenic pressures. In long term, this could mean the extinction of the species. We investigated the dynamics of occurrence and distribution of three canid species (*Atelocynus microtis*, *Cerdocyon thous*, and *Spheotos venaticus*) considering human threats to their habitats in the Amazon Rainforest. We analyzed the environmental thresholds for the occurrence of these species and related to the future projections of climatic niches for each one. All three species will be negatively affected by climate change in the future, with losses of up to 91% of the suitable area of occurrence in the Brazilian Amazon. *A. microtis* appear to be more forest‐dependent and must rely on the goodwill of decision‐makers to be maintained in the future. For *C. thous* and *S. venaticus*, climatic variables and those associated with anthropogenic disturbances that modulate their niches today may not act the same way in the future. Even though *C. thous* is least dependent on the Amazon Forest; this species may be affected in the future due to the ecological traps. *S. venaticus*, can also undergo the same process, but perhaps more drastically due to the lower ecological plasticity of this species compared to *C. thous*. Our results suggest that the ecological traps may put these two species at risk in the future. Using the canid species as a model, we had the opportunity to investigate these ecological effects that can affect a large part of the Amazonian fauna in the current scenario. Considering the high degree of environmental degradation and deforestation in the Amazon Rainforest, the theory of ecological traps must be discussed at the same level as the habitat loss, considering the strategies for preserving the Amazon biodiversity.

## Graphical abstract

Considering the evolutionary history of the species and the rapid changes in land use in the Amazon Forest, we suggest that anthropogenic environments may provide false clues for species adaptation, which could lead to an ecological trap in the future. For at least two of the three canid species studied, climatic variables and those associated with anthropogenic disturbances that modulate their niches today may not act in the same way in the future. Thus, rapid environmental changes can act as ecological traps for these species and, in the future, they may no longer survive in most areas considered suitable for them today. Using canid species as a model, we investigated ecological effects that could affect a large part of the Amazonian fauna in the current and future scenario.

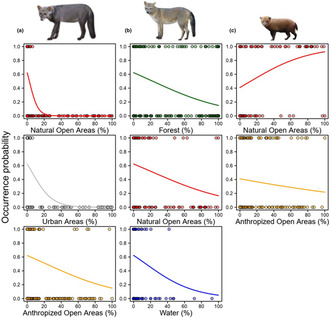

## 1 INTRODUCTION

The Amazon is home to thousands of species of terrestrial vertebrates, which interact with each other and with the environment in a unique way. These interactions define the ecological niches of these species and how they are distributed in time and space (Hirzel & Le Lay, 2008). Deforestation, climate change, and other anthropogenic pressures have caused drastic changes in the structure of native habitats in the Amazon in the last 40 years (PRODES, 2022), which have consequences on the interactions of species with their environment (Coelho et al., 2014; Mendes‐Oliveira et al., 2017; Sales et al., 2019). Deforestation and climate change are factors that already act concurrently, transforming natural habitats into degraded areas in the Amazon, where the drought‐deforestation feedback will leave the climate in these areas warmer and drier (Staal et al., 2020).

After 10 years of reduction in deforestation (2004–2014), the Brazilian Amazon has experienced an increase in degradation. During the last 3 years (2019–2022), about 32.500 km^2^ of the Brazilian Amazon Forest were deforested (PRODES‐Terrabrasilis/INPE, 2022). About 65% of this deforestation was concentrated in the States of Pará and Mato Grosso, which border the Cerrado Biome, also is called the Brazilian savannah. This entire frontier area of the Brazilian Amazon is called the Arc of Deforestation, as it concentrates about 90% of the region's total deforestation (PRODES‐Terrabrasilis/INPE, 2022). Significant forest losses and climatic variations are projected for this region in the coming years, increasing the transition zone area between forests and savannah (Lyra et al., 2016). These changes can negatively affect specialist forest species, and in turn favor the use of the degraded landscape by species able to use savannah habitats (Sales et al., 2020). Nevertheless, degraded landscapes can lead species into ecological traps, where modified environments may lead to false clues to favorable habitats, making it impossible to accurately assess the suitability value for species in these habitats (Hale & Swearer, 2016).

Ecological traps occur when species settle in lower‐quality habitats, even if this reduces their productivity or survival (Hale & Swearer, 2016). This situation has been happening mainly due to the rapid environmental changes caused by anthropogenic actions, which generate new conditions in the habitats, different from the original ones, but which may be somehow related to the evolutionary history of the species (Robertson et al., 2013). In this way, species capture signals, that are not indicative of habitat quality, and mistakenly establish themselves in unfavorable locations for their survival in the medium and long term (Robertson & Hutto, 2006). In this paper, we raise the possibility that this process occurs with some species in the Amazon region, driven by the intense dynamics of land use and rapid environmental changes caused by degradation, deforestation, and climate change. In this way, understanding the factors that determine the occupation of high‐quality sites by species can help to mitigate the harmful effects of choosing poorly adapted habitats (Gilroy & Sutherland, 2007; Robertson & Hutto, 2006).

Most neotropical vertebrates live in natural environments already altered at some level, directly or indirectly, by human activities. The response of the fauna to significant changes in their native habitats can vary according to their ecological demands and niche differences (Hagen et al., 2012; Sih et al., 2011). Also, a species' fitness is closely related to its evolutionary history, shaped by environmental factors that influenced the ecological preferences and demands of the species over an evolutionary time (Harper et al., 1961). In this paper, we investigate how recent significant changes in natural environments may alter patterns shaped during evolution and whether species' responses can mislead them into ecological traps and thereby reduce their fitness. In this context, it was necessary to have a basic knowledge about the original distribution of the species, habitat preferences, and what determines their occurrences.

Members of the Canidae family have undergone morphological and behavioral changes due to environmental changes and degradation of their native habitats (de Moura Bubadué et al., 2015; Figueirido et al., 2015; Yom‐Tov et al., 2007). Canids appeared around 40_Ma in North America (Wang et al., 2007). They diversified throughout the Pleistocene and occupied different niches and geographical locations (Berta, 1987; Tchaicka et al., 2016). The emergence of a wide variety of morphologies and diets over a relatively short evolutionary period (Figueirido et al., 2015; Nyakatura & Bininda‐Emonds, 2012; Perini et al., 2010), make canids a very interesting group for studies of ecological plasticity. For instance, small variations in masticatory or locomotor systems have caused large differences in their adaptive capacity (Figueirido et al., 2015; Penrose, 2019). Currently, 10 species of canids occur in South America, with high morphological and ecological diversity among them (Perini et al., 2010).

**FIGURE A1:**
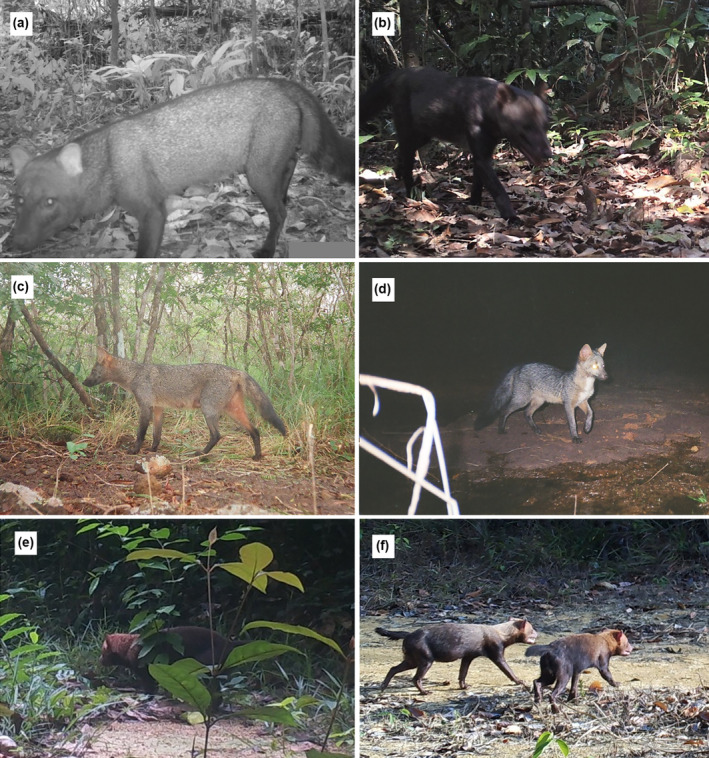
Photographs of camera traps of wild dog recorded in the Amazon. (a) *Atelocynus microtis*, photograph captured at FLONA Caxiuanã, Pará‐Brazil (TEAM‐Caxiuanã/Fernanda Santos); (b) *Atelocynus microtis*, photograph captured at ESEC Terra do Meio, Pará‐Brazil (Monitora ICMBIO/Cintia Lopes); (c, d) *Cerdocyon thous*, photograph captured at FLONA Carajás, Pará‐Brazil (Ana Cristina Mendes‐Oliveira); (e) *Spheotos venaticus*, photograph captured at CKBV's Company area, Paragominas, Pará‐Brazil (Ana Cristina Mendes‐Oliveira); (f) *Spheotos venaticus*, photograph captured at HYDRO Company area, Paragominas, Pará‐Brazil (Ana Cristina Mendes‐Oliveira and Øystein Wiig).

Three species of canids are represented in the Amazon: *Atelocynus microtis* (Sclater, 1883) (short‐eared dog), *Speothos venaticus (Lund, 1842)*, (bush dog), and *Cerdocyon thous* (Linnaeus, 1766) (crab‐eating fox) (Figure A1). These are small to medium‐sized (from 4 to 11 kg) canids with a broad and partly sympatric distribution in the Amazon region. The occurrence and distribution of canids have been associated with environmental characteristics and interspecific competition (de Moura Bubadué et al., 2015; de Oliveira & Pereira, 2013; Rocha et al., 2020).

In this paper, we investigate the dynamics of occurrence and distribution of *A. microtis*, *C. thous*, and *S. venaticus* related to anthropogenic threats imposed on their habitats in the Amazon Forest. Using two modeling scales, we investigated the possible environmental thresholds for the occurrence of the three species, how their distribution might be influenced by climate change and deforestation, and whether these factors can lead these species to fall into ecological traps in the Amazon Rainforest. For that, we used climatic niche modeling of species for all of South America and modeling of the environmental factors that determine the occurrence of these species, especially in Brazil. We also model the future occurrence and distribution of those species, considering a pessimistic climate change scenario. Verifying the influence of environmental variables on species occurrence and contrasting their ecological requirements with the pattern of loss of area of climatic suitability, we inferred about the future of these species in the light of the ecological traps' theory. Our hypothesis is that all species are influenced by different environmental variables, and changes in these variables can lead species to ecological traps.

## 2 METHODS

### 2.1 The target species

*A. microtis* (Figure A1a,b) is the only endemic species in the Amazon Biome with a wide distribution in the region, extending from Ecuador to Brazil (Leite‐Pitman & Williams, 2011). Rocha et al. (2020) showed a strong connection of *A. microtis* to forest habitats, and how it has been negatively affected by deforestation in the Amazon. There are few records of the species over the last 30 years (Peres, 1991; Koester et al., 2008; Michalski, 2010; Blake et al., 2012; Ayure & González‐Maya, 2014). However, this species seems to have been more frequently recorded previously (Grimwood, 1969). Possible reasons for its apparent reduced population size are infection from diseases acquired through contact with domestic dogs and the loss of habitat (Leite‐Pitman & Williams, 2011). *A. microtis* is a mesocarnivorous canid with a generalist carnivore diet but also including fruit (Blake et al., 2012; Penrose, 2019; Pitman & Williams, 2004). It is solitary (Peres, 1991), usually with a diurnal activity (Pitman & Williams, 2004; Blake et al., 2012). Individuals of this species do not seem to tolerate disturbed habitats and prefer continuous primary lowland and upland forests (Leite‐Pitman & Williams, 2011; Michalski, 2010; Rocha et al., 2020). This species is classified as “Near Threatened” in the IUCN Red List of Threatened Species (Leite‐Pitman & Williams, 2011).

*Cerdocyon thous* (Figure A1c,d) has the smallest distribution area in the Amazon Biome of the three species. However, it is widely distributed in the rest of South America. It is common in the Pantanal (Brazilian Swamp), Atlantic Forest, Cerrado (Brazilian Savannah), Amazon, and Caatinga (Brazilian dry forest) biomes, and frequently recorded at forest edges (Brady, 1979; Courtenay & Maffei, 2004). Individuals of this species have twilight and nocturnal habits, are solitary or live in small groups (Macdonald & Courtenay, 1996). Their diet is considered omnivorous, generalist, and opportunistic, feeding on invertebrates, small vertebrates, and even carrion, but they also frequently feed on fruits (Bisbal & Ojasti, 1980; Macdonald & Courtenay, 1996). Of the three species, *C. thous* can be considered the one that least depends on the forest, and has also adapted to open environments, including human‐made areas (Faria‐Corrêa et al., 2009; Ferraz et al., 2010). This species is classified as “Least Concern” in the IUCN Red List (Lucherini, 2015).

*Speothos venaticus* (Figure A1e,f) is the canid with the broadest distribution in the Amazon, although it is not as common to register this species as *C. thous* (de Oliveira et al., 2018; DeMatteo & Loiselle, 2008; Guimarães et al., 2015). In Brazil, this animal occurs in the Amazon, Pantanal, Atlantic Forest, Cerrado, and Caatinga biomes, especially in humid forests and preserved riparian forests. Despite this, the species can occur in degraded areas (DeMatteo & Loiselle, 2008; Lima et al., 2014; Silveira et al., 1998), it is predominantly found in preserved areas (DeMatteo & Loiselle, 2008; Michalski, 2010), but also was confirmed in native environments in the Cerrado of central‐west of Brazil (Lima et al., 2014). The authors demonstrated the preference of this species for native environments instead of plantation areas (Lima et al., 2014). Also, most records of the species in cultivated lands indicated a rapid passage through these areas, while in the forest and savannah areas, they remained foraging, eating, or resting. *S. venaticus* can be considered a hypercarnivore, feeding on small vertebrates and larger mammals such as pacas, agoutis, and even capybaras (Beisiegel & Ades, 2004; Zuercher et al., 2005). They are social animals, which may have a cooperative hunting system, but lone individuals are also frequently observed (Beisiegel & Ades, 2004; DeMatteo et al., 2011). This species is classified as “Near Threatened” in the IUCN Red List (DeMatteo et al., 2011). Its greatest threat appears to be habitat degradation.

### 2.2 Species occurrence data

**FIGURE 1:**
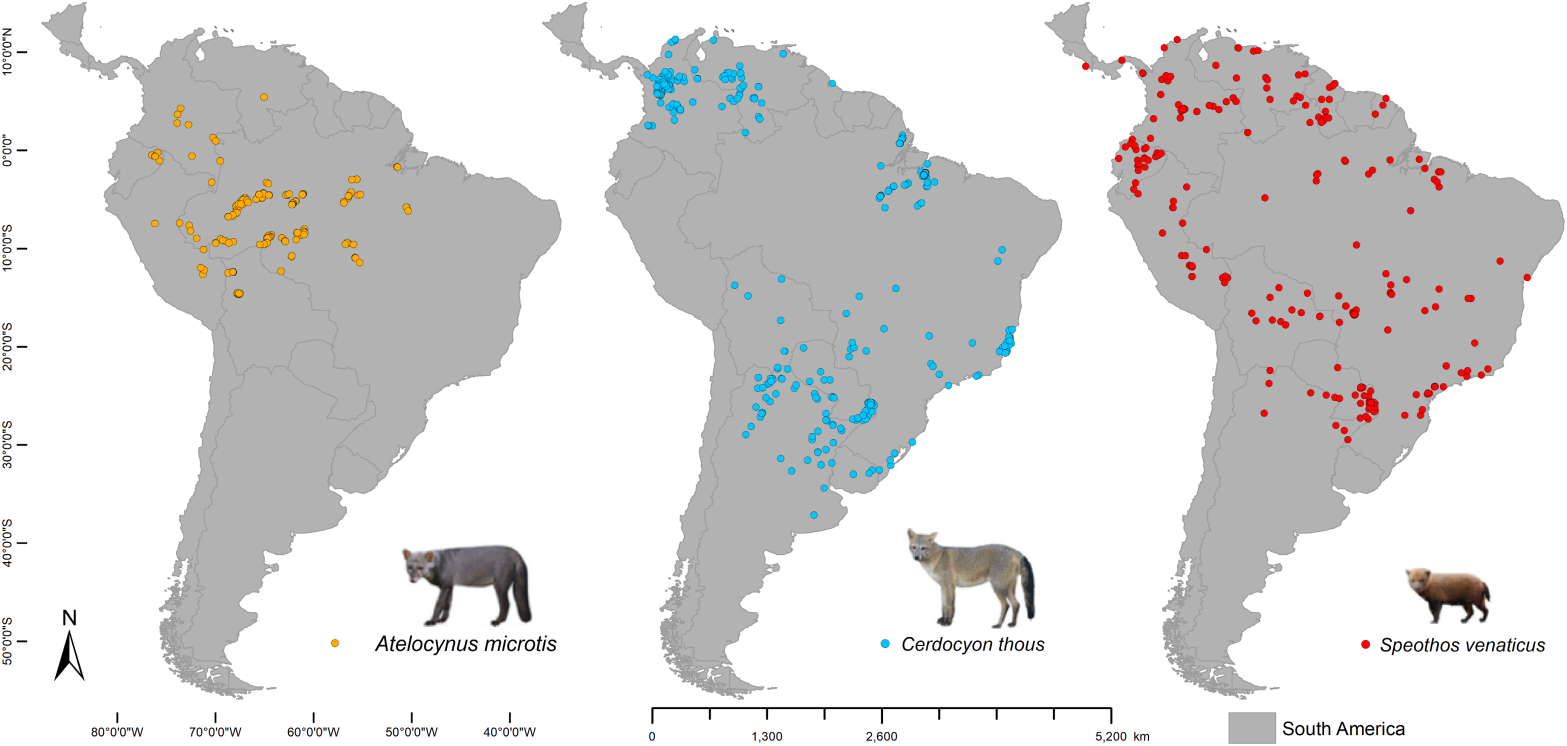
Distribution of occurrence records of the three species of Amazonian wild canids in South America. Data obtained through literature, databases, and field inventories.

The study area encompasses the whole Amazon Biome, also known as the Amazon Rainforest, which constitutes the largest tropical forest in the world, covering nine countries in South America: Bolivia, Brazil, Colombia, Ecuador, French Guyana, Guyana, Peru, Suriname, and Venezuela (IBGE, 2004; Junk et al., 2011). The species distribution database was built considering the whole of South America so that the potential distribution models covered the entire range of the species studied, despite the discussion is focused on the Amazon Biome (Figure 1).

The database of geographic records of the three species was built from literature data and online databases, such as Global Biodiversity Information Facility (GBIF; http://www.gbif.org/; GBIF Occurrence Download https://doi.org/10.15468/dl.dyy7qk) and Species Link (http://splink.cria.org.br/; Figure 1; Table S1). Especially for *A. microtis*, we used most of the records from the Rocha et al. (2020) publication. The database published by Nagy‐Reis et al. (2020) (Available at https://esajournals.onlinelibrary.wiley.com/doi/10.1002/ecy.3128) was also consulted. In addition, we used data from inventories of camera traps and Linear Transection census, carried out by the team of the Laboratory of Ecology and Zoology of Vertebrates of the Federal University of Pará (Brazil), between 2000 and 2019 (Figure A1; Table S1).

We searched for geographic information on the rare localities and municipalities whose coordinates were unavailable using Google Earth v7.1.2 (https://www.google.com/earth/) and online gazetteers (e.g., directory of cities and towns in the world; www.fallingrain.com/world). Using these same resources, we checked all records with coordinates to ensure the best reliability of the occurrences used in this study. We verified the veracity of the points contrasting with what is already known about each species' distribution. Some doubts were solved by the researchers who collected the data. In addition, points that did not have precise information on geographic coordinates, or corresponded to questionable places, such as rivers or lakes, were discarded. Also, duplicated coordinates recorded in different databases were discarded. The remaining records (Table S1) were used for niche modeling.

We used two sets of models to investigate the dynamics of occurrence and distribution of the three wild canids related to human threats imposed on their habitats in the Amazon Rainforest. The first considers the climatic variables for modeling the species niche for all of South America. Moreover, the second set of Generalized Linear Models includes the environmental factors that determine the occurrence of each of these canid species.

### 2.3 Climatic variables and niche modeling

To generate the distribution predictions for the three canid species, we gathered 19 climatic variables (Hijmans et al., 2005), available for the current, and future projections of the SSP585 climate scenario from global climate models (GCM's: BCC‐CSM2‐MR; CanESM5; IPSL‐CM6A‐LR; MIROC‐ES2L; and MIROC6), selected by a cluster analysis (Varela et al., 2015) in the WorldClim v2.1 database. We limited the variables to the South American continent, with a grid resolution of 0.041° at the Equator (~4 km or 2.5 arc‐min).

We standardized all variables, so they had their means equal to 0 and their variance equal to 1. To avoid collinearity between climate variables, we performed a Principal Component Analysis (PCA) to reduce the dimensions of bioclimatic variables (De Marco & Nóbrega, 2018). We selected the first six PCs from those, responsible for ~97% of the original raw climatic variance of the predictor variables (Table S2). These independent variables allowed us to produce more reliable predictions for the species distributions and avoid model overfitting (Jiménez‐Valverde et al., 2011). To maintain the dimensionality of the climate data over time, we used the coefficients obtained from the PCA performed with current climate data to compute the scores for future climate data for each selected AOGCM (Sillero & Márcia Barbosa, 2021).

We partitioned the three species occurrences using a checkerboard pattern (Muscarella et al., 2014; Roberts et al., 2017) with an aggregation factor of two. According to this partitioning pattern, we divided the species occurrences into two subsets, geographically structured as a checkerboard table. One subset was used to test the models on a first run, while the other was used to evaluate the models. On a second run, these subsets were inverted, and the one used to evaluate the models was used to test, and the one used to test was used to evaluate the models. We always consider the proportion of subsets with 30% of data for testing and 70% for training.

We considered three modeling methods to produce our models: (1) Random Forest (RDF) statistical method, and two machine learning methods, (2) MaxEnt (MAX), and (3) Support Vector Machines (SVM). We trained the models for the three species considering the ecoregions shapefile provided by the World Wildlife Fund website (https://www.worldwildlife.org/biomes), restricting the climatic variables to ecoregions with known occurrences for the species. This procedure is essential to delimit the M section of the Biotic–Abiotic–Migration diagram (Barve et al., 2011; Saupe et al., 2012; Soberón, 2007; Soberón & Peterson, 2005) and improve the model predictions for the focus species.

We made use of pseudo‐absences to train the models. Therefore, we restricted their allocation in the geographic space after they were environmentally restricted, considering an environmental space based on a climatic environment (Lobo & Tognelli, 2011; VanDerWal et al., 2009).

To evaluate our models, we considered the Jaccard's similarity index proposed by Leroy et al. (2018), which varies from 0 to 1, where around zero values indicate no‐better‐than‐random predictions, while values higher than 0.5 are considered as acceptable predictions. Different from other metrics commonly used in distribution modeling studies [e.g., the area under the curve (Fielding & Bell, 1997) or accurate skill statistic (Allouche et al., 2006)], the Jaccard index is both prevalent and pseudo‐absence independent, yielding less uncertain metrics for the models.

**FIGURE 2:**
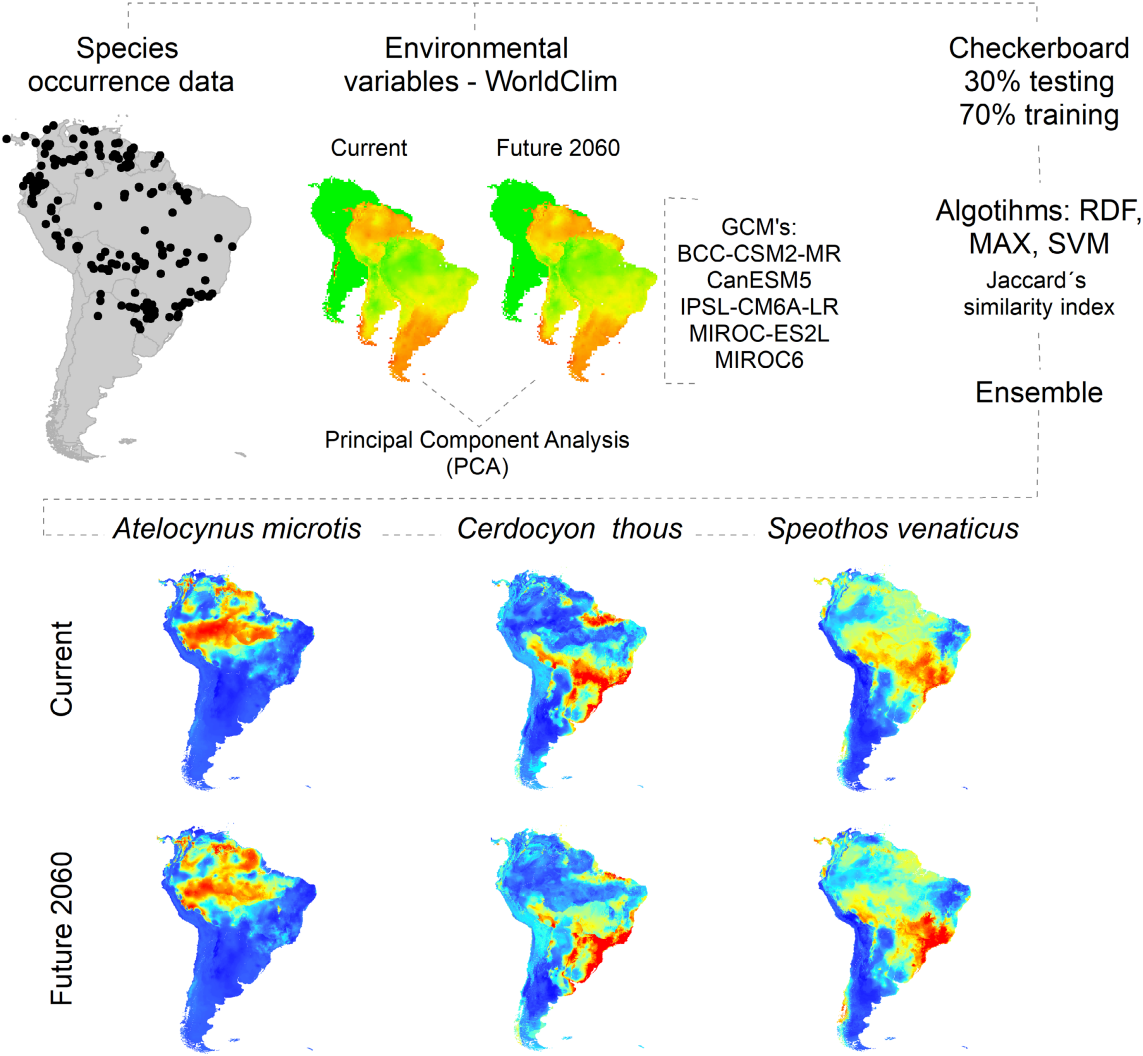
Methodological procedure for obtaining Niche Modeling, using climate variables for the current and future period (2060), configuration and evaluation of ENM available in the ENMTML package.

To cut the suitability matrices into presence/absence matrices, we considered the threshold that maximized the Jaccard index. Finally, we used a mean ensemble weighted by the best Jaccard values in each modeling method in order to produce the species final distribution range (Figure 2). We perform the modeling by the package ENMTML (Andrade et al., 2020) in *software* R (v4.0).

### 2.4 Environmental factors determining the occurrence of the canids

**FIGURE 3:**
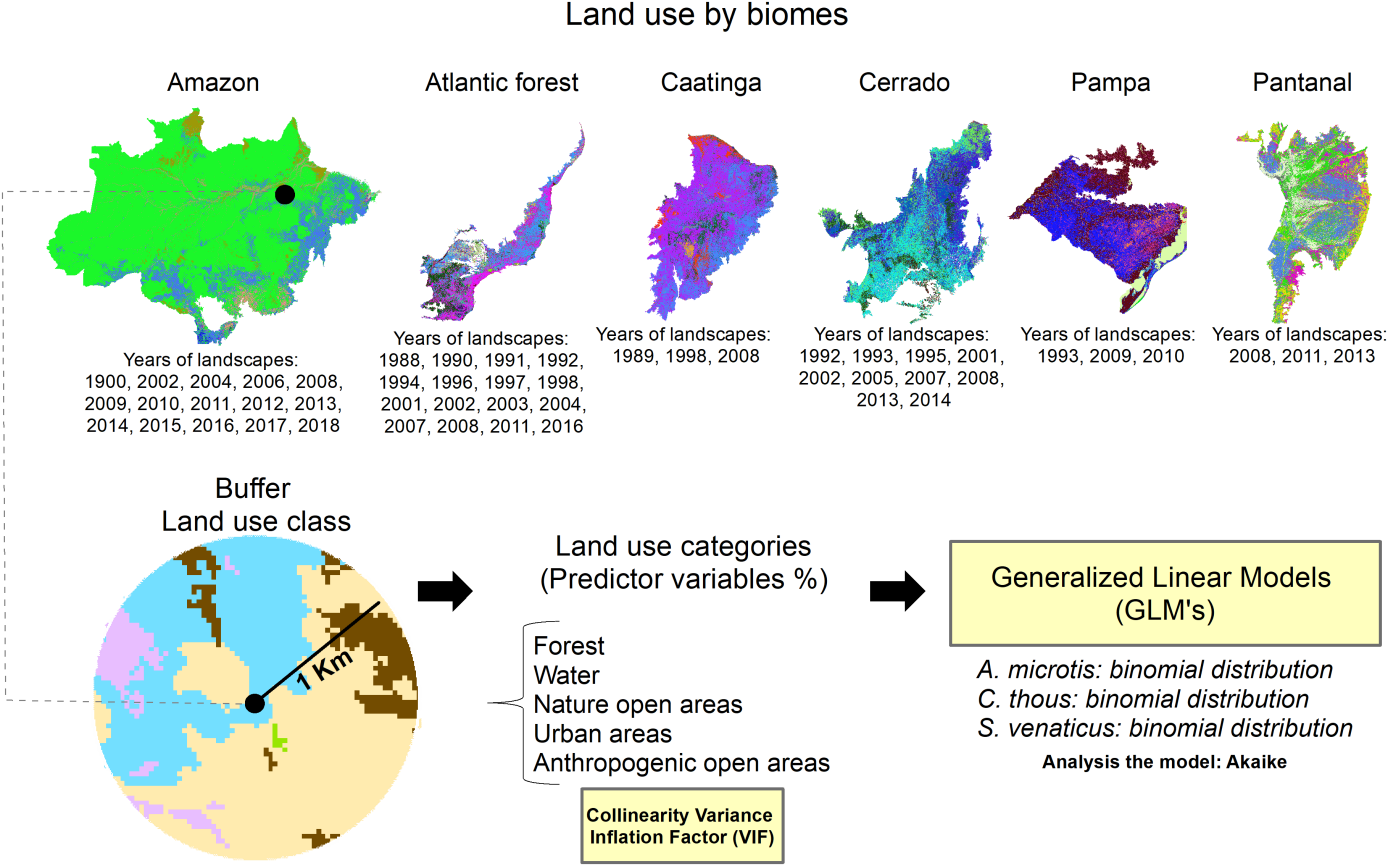
Methodological procedure used to obtain the environmental factors that determine the occurrence of *Atelocynus microtis*, *Cerdocyon thous*, and *Speothos venaticus*, through the analysis of Generalized Linear Models (GLMs). The first line of maps shows the Brazilian biomes and the years of extraction of environmental variables according to the year of species records obtained for each biome. The square in the lower left corner shows the 1 km buffer established for the extraction of land‐use variables for each recording point of the canid species. The colors in the buffer are for illustrative purposes only, representing different land‐use categories. The circle highlights the land‐use categories extracted to be used in the Generalized Linear Models. The first “Land use category” list cites all the variables used in the VIF analysis to test the multicollinearity. The second list of Generalized Linear Models cites the species and the respective families used in the modeling for each of them. The third list, “Predictors variables,” presents the variables used in the GLM's models.

We only used the records occurring within Brazil to analyze which environmental variables and landscape structures influenced each species' occurrence. We limited it to Brazilian points because we used the Landsat classified image database from MapBiomas. v5.0 collection, which is available just for this country (https://mapbiomas.org/). In the GeoTiff scenario, this database presents the annual mapping of land cover and land use in Brazil between the historical period of 1985 to 2019 (Souza et al., 2020). We listed the coordinates of each occurrence of *A. microtis*, with information on scenarios in the Amazon Biome, from 2002 to 2019, a period that includes all records of occurrences of this species. We did the same thing for *C. thous*, using data from 1988 to 2016, including the Amazon Forest, Caatinga, Cerrado, Atlantic Forest, Pantanal, and Pampas biomes. For *S. venaticus*, we used data from 1900 to 2017, including the following biomes: Amazon, Cerrado, Caatinga, Atlantic Forest, and Pantanal (Figure 3; Table S1).

We defined a buffer for each coordinate of occurrence for each species, with a radius of 1 km, from which we extracted information on land use (Teixeira‐Santos et al., 2020). We obtained the pixel values and land‐use class codes in each buffer (available at: https://mapbiomas.org/downloads_codigos) and transformed these values into categories of percentages. Considering a buffer of a 1 km radius, we selected occurrence points with less than a 20% overlap between the buffers, defining an approximate minimum distance of 2 km between each occurrence point. All points that exceeded a 20% buffer overlap were discarded. The distance of 2 km was defined based on the home range of *C. thous*, which is the species with the greatest overlapping of occurrence points (Brady, 1978; Macdonald & Courtenay, 1996). After eliminating buffers with overlapping criteria, we selected 277 independent records with a minimum distance of approximately 2 km between them, totalizing 64 records of *A. microtis*, 63 of *C. thous* and 100 of *S. venaticus*.

About 16 land‐use classes were recorded in the occurrence scenarios of the buffers. We grouped together some classes with similar landscape effects. For example, we grouped together all the forest categories under “Forest,” also the types of rivers, lakes, and other watercourses were grouped together under “Water.” In contrast, pasture, agriculture, and similar activities were grouped together under “ Anthropized open areas.” In addition, other classes with a frequency of less than three occurrence points, such as “mining,” were discarded (Table S3). In the end, we considered five significant land‐use categories to assess the environmental factors that can determine the occurrence of species. They are: (Forest) Percentage of forest cover, which includes all native forest formations of the Amazon; (Water) Percentage of water bodies, including streams, rivers, lakes, and non‐forest natural wetlands; (Nat_open_areas) Percentage of natural open areas, including savannas, grasslands and rocky outcrops; (Urban_areas) Percentage of urban infrastructure; and (Anthr_open_areas) Percentage of anthropized open areas, including pasture, perennial annual crop, agriculture, and pasture mosaic (Figure 3; Table S3).

To verify whether the covariates present collinearity, we calculated the variance inflation factor (VIF) for each full model of the probability of occurrence of canids. This way, we dropped the covariate with high VIF values and recalculated the VIFs until all values VIFs of each covariable were lower than 6 (Table S4; Zuur et al., 2010). With the collinearity problem removed, we built generalized linear models (GLMs) to assess how different landscape aspects affect the occurrence probability of *A. microtis*, *C. thous*, and *S. venaticus*. All final models (i.e., those more parameterized and without multicollinearity, Table S4) were submitted for residual analyses to assess the adequacy of error distribution and overdispersion using DHARMa package R (Hartig, 2020). All the models showed suitable dispersion parameters (Table S4; Figure S1). We submitted the final models to the model selection approach using the “dredge” function and subsequent application of the model averaging using the “model.avg” function to calculate the relative importance value (RIV) of each predictor on the model using the “sw” function. The RVI means the sum of the Akaike weights (i.e., probability of a model to be the most plausible one) for the models in which each predictor appears. We based the RVI outputs on the best ranked models with ΔAICc < 4, because it is considered a conservative value to calculate the parameter weights (Solar et al., 2016; Vierling et al., 2013). The RIV can be considered the general support for each predictor variable in all models. The 70% cutoff threshold is arbitrary and was defined to differentiate important and unimportant predictors (Burnham et al., 2002; Deschutter et al., 2017; Everaert et al., 2018; Terrer et al., 2016). We extracted those variables' beta values (slopes) with an RVI > 0.70, based on the estimates of the model averaging (coefficients of the total average), shown in the graphs.

## 3 RESULTS

### 3.1 Climatic variables and niche modeling

Overall, we gathered 319 records of *A. microtis*, 691 of *C. thous* and 271 of *S. venaticus*, throughout South America (Table S1). In general, our species niche distribution models reached adequate values for the Jaccard index (0.612 average), reaching higher predictive values than those expected by chance. All models generated from the three species and the ranges of suitability of their respective algorithms can be found in the Figure S2.

**FIGURE 4:**
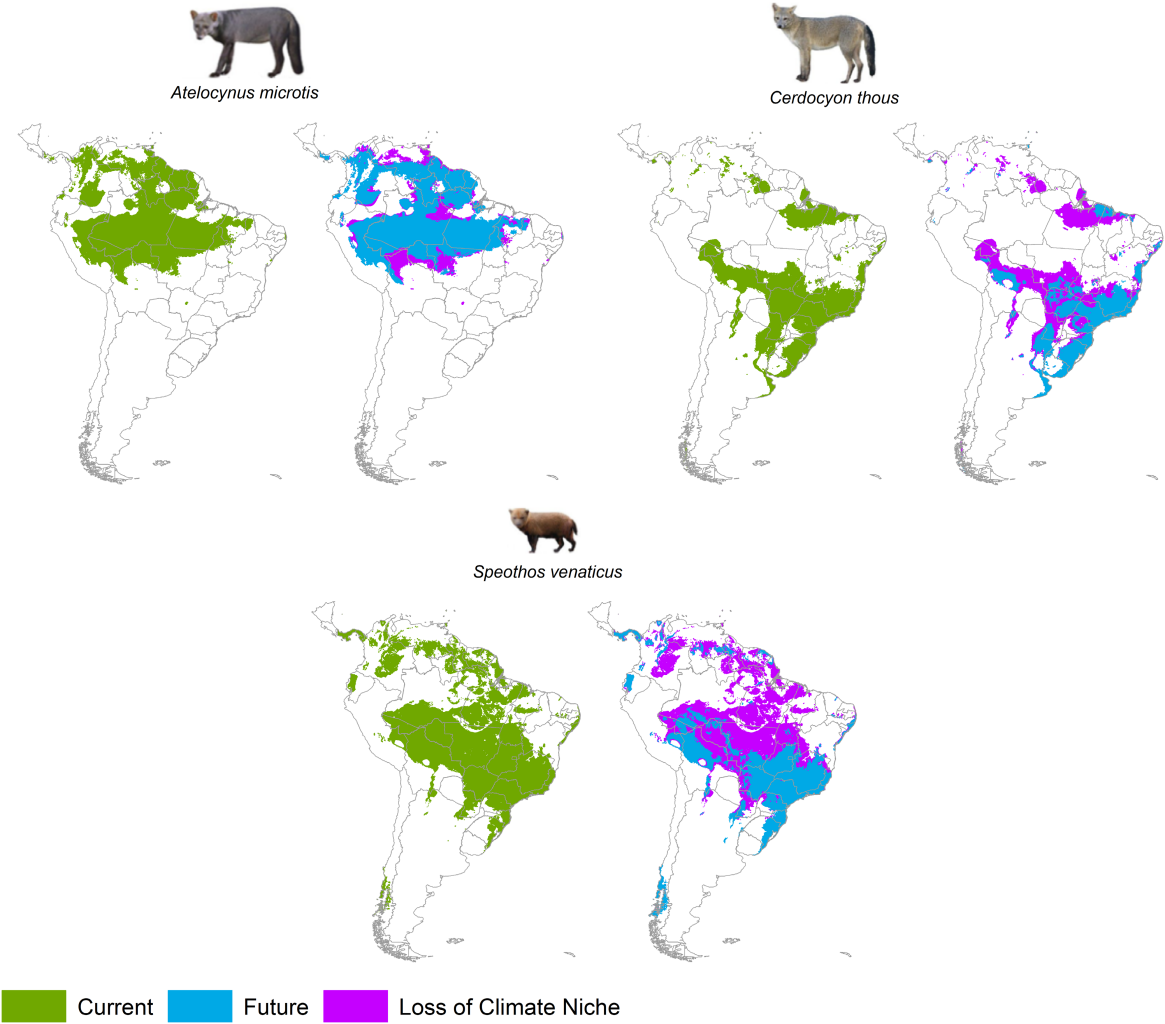
Predictions of climatic niche distributions for the three species of wild Amazon canids (*Atelocynus microtis*, *Cerdocyon thous*, and *Speothos venaticus*) in South America, based on a mean ensemble considering the best Jaccard values for each modeling method. Current—Areas of occurrence considering the current climate suitability for the species; Future—Climate niche projections for 2060 for each species; Loss of Climate Niche—Areas that will lose climate suitability for the species in the future.

Considering the final weighted ensemble for the three species (Figure 4), *A. microtis* in both current and future scenarios was more restricted to the western part of the Amazon Forest, including Colombia, Peru, Bolivia, and also in the northern part, including Venezuela, Suriname, Guyana, and French Guyana. In addition, we found areas of suitability for this species in the extreme northeast of the Brazilian Amazon, which includes the west of Amapá State and further north, in the south of Guyana near the northeast of the state of Roraima (Brazil). We observed a loss of 18.43% of the total *A. microtis* climate suitability area in South America in the future. Of this total loss, about 15% is supposed to occur in the Brazilian Amazon. In this region, these losses are distributed mainly in areas with high anthropic pressure, close to the southern and southeastern limits of the Forest, in a transition zone with the Cerrado, in the states of Rondônia and Mato Grosso. However, we also observed areas of loss concentrated in the eastern borders of the species distribution, which are areas of high anthropic pressure in central Amazonia (Figure 4).

*Cerdocyon thous* has high climatic suitability in open areas and anthropogenic landscapes, inside and outside the Amazon Biome, in both current and future scenarios. Its potential distribution is within the Amazon natural savannahs in eastern Amapá state and part of Roraima state in northern Brazil. The species also shows suitability in anthropized areas in the southern limits of the Amazon region, and in the transition zone with the Cerrado Biome, especially in the states of Mato Grosso and Rondônia (Figure 4). In the current scenario, the area of climatic suitability includes a strip in the northeast of the Brazilian Amazon, where high anthropogenic pressure is concentrated in the region. Outside the Amazon, this species also had climatic suitability for natural areas of Cerrado and anthropized open areas in the central‐western, southeastern, and southern areas of Brazil east‐central Bolivia, southeastern Paraguay, Uruguay, and northeast of Argentina. The future projected ensemble model predicts the loss of 56.54% of the *C. thous* climate suitability area across South America (Figure 4). About 88% of this total loss could occur in the Brazilian Amazon, precisely in the areas of most significant anthropic pressure, including the northern portion of the state of Pará and in the southern and southwestern limits of the Brazilian Amazon.

The projected area of climatic suitability for *S. venaticus* is almost as wide as for *C. thous* (Figure 4). However, the current distribution of *S. venaticus* includes both areas of Cerrado and dense forests, but also other Brazilian biomes and anthropogenic open areas (Figure 4). When we analyze the projection for the future, *S. venaticus* is the species that loses the most area with climatic suitability, among the three canids, with a reduction of 57.18% for all of South America. However, about 91% of this total loss will occur in the Brazilian Amazon (Figure 4). Part of this loss in the Amazon region is concentrated in naturally open areas such as eastern Amapá State and northeast Roraima State, southeast Venezuela and southwest Guyana. Large patches of Amazonian Savannah characterize these areas. On the other hand, most of the potential loss of area of climatic suitability for *S. venaticus* in the future overlaps with regions of more significant anthropic pressure in the Amazon, which includes the entire area of the “Arc of Deforestation.”

### 3.2 Environmental factors determining the occurrence of the canids

According to the relative importance value extracted from the model‐averaging subset with ΔAICc<4, we observed that each variable influenced the occurrences of species differently, according to their ecological requirements (Table S5). The beta values (estimates parameters) from the most parsimonious models (ΔAICc<4) can be seen in the Table S6.

**FIGURE 5:**
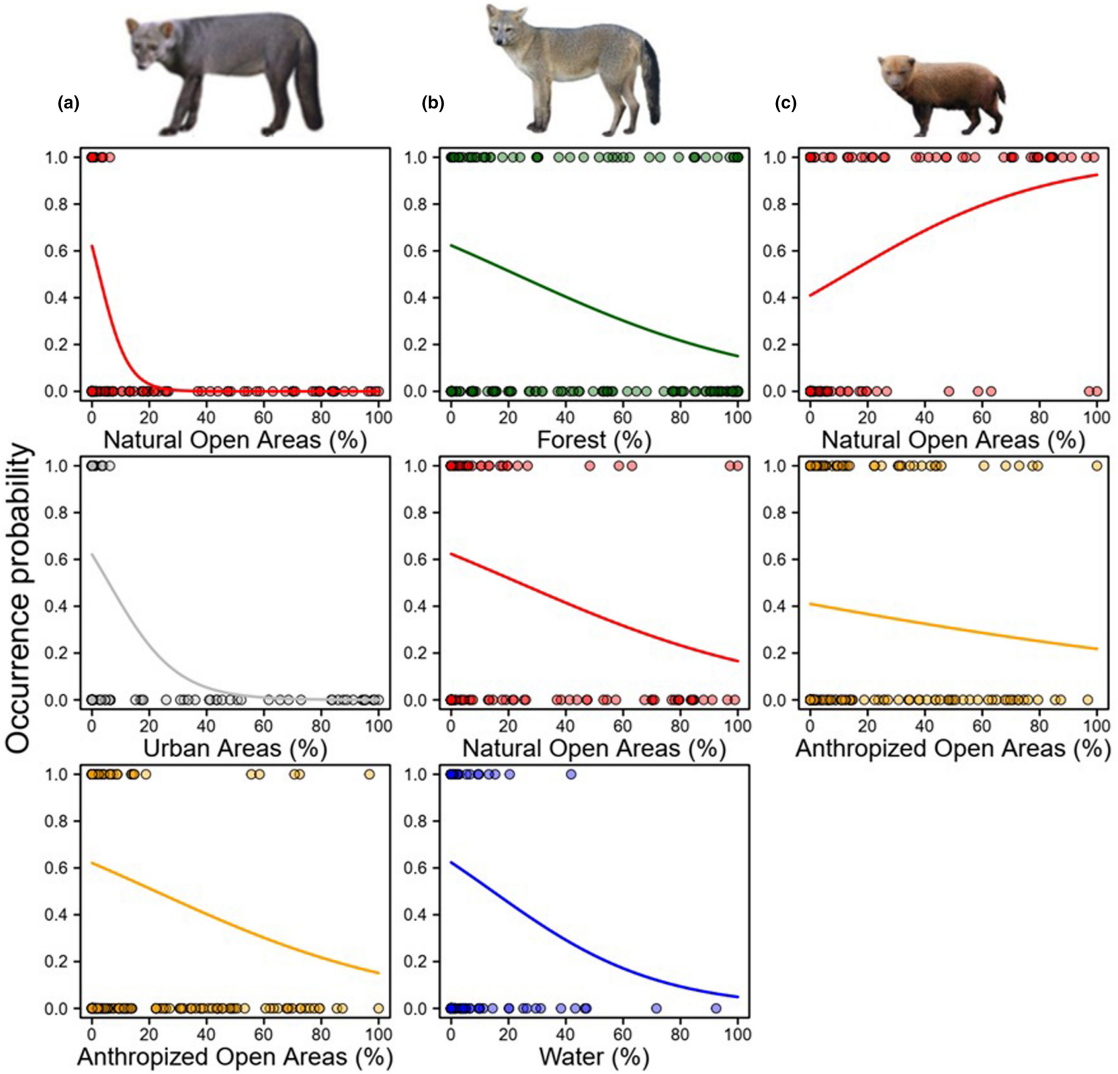
Relationships of each selected predictor variable with strong influence (w+ >0.70, see Table S5) on the occurrence of canid species in the Amazon. (a) *Atelocynus microtis*, (b) *Cerdocyon thous* and (c) *Speothos venaticus*. The graphs were based on model‐averaging estimates of the model's subset with ΔAICc<4.

For *A. microtis*, the percentage of natural open areas, urban areas and open areas anthropized were the variables that had a negative influence on the occurrence of species, showing an exponential decrease in the probability of occurrence of this species with the increase in the percentage of these variables (Figure 5a; Table S5) The variable % forests did not reach a threshold for selection and water showed little importance for the occurrence of this species (Table S5).

For *C. thous*, the percentage of the forest, natural open areas and water in the landscape had negative effects on the probability of occurrence, showing a relationship in the decrease of the probability of occurrence of the species in areas with higher percentages of these variables (Figure 5b; Table S5). For this species, all native habitat variables had a negative influence (Figure 5b; Table S5).

Last, for *S. venaticus*, only the percentage of open natural areas proved to be a variable with a strong positive influence on the probability of occurrence of this species (Figure 5C; Table S5). The positive relationship shows that the greater the percentage of open natural areas, the greater the probability of occurrence of the species (Figure 5C; Table S5). However, the percentage of anthropized open areas had a negative effect on the probability of occurrence of *S. venaticus* (Figure 5C).

## 4 DISCUSSION

Our results show that the occurrence of the three Amazonian canid species *C. thous*, *S. venaticus* and *A. microtis* may be negatively affected by climate change in the future. The loss of suitability range goes from 15% to 91% in South America, and from 18% to 56% in the Brazilian Amazon. However, the effects can be differentiated between the species according to their ecological demands and the environmental variables that modulate their climatic niches. For *C. thous* and *S. venaticus*, the climate variables and those associated with anthropogenic disturbances that modulate their niches today, may not act the same way in the future. Thus, the rapid environmental changes might function as ecological traps for these species (Robertson & Hutto, 2006), and in the future they may no longer survive in most of the areas considered suitable for them today.

Among the three species *A. microtis* has the most restricted distribution confined to the Amazon Forest and the one with the least projected loss of suitable climatic areas in the future. This species is a specialist in forest habitats, in particular in continuous and preserved forests (Leite‐Pitman & Williams, 2011; Michalski, 2010). Although our results do not show the direct importance of the percentage of forest on this species, all other variables of open and anthropic areas had a negative influence on the probability of occurrence of *A. microtis*. In this way, converting forests into anthropized or urbanized open areas may reduce the occurrence of this species. Furthermore, we observed that the loss of suitability of occurrence of *A. microtis* in the Amazon coincides with areas of high pressure of land‐use conversion. Rocha et al. (2020) also demonstrated that this species' fitness loss is directly related to forest loss due to deforestation and forest degradation. In addition to the distribution of the climatic niche of this species coinciding mainly with the most conserved regions of the Amazon Forest, our results indirectly corroborate the connection of this species with forested areas. Although we have not demonstrated the significant influences of watercourses on the running of *A. microtis*, this species has interdigital membranes that may be an adaptation to move in soft soil (Leite‐Pitman & Williams, 2004; Castelló & Sillero‐Zubiri, 2018). Thus, the future loss of areas suitable for *A. microtis* cannot be related to the ecological trap theory. This species will likely no longer occur in unfavorable habitats due to its aptitude for higher‐quality forest habitats.

For *A. microtis*, one of the consequences of deforestation and human pressure is increased hunting (Constantino, 2016), including hunting with domestic dogs. The disease transmission by domestic dogs can pose a threat to *A. microtis* (Leite‐Pitman & Williams, 2011; Schenck & Staib, 2004). Our results corroborate with those of Rocha et al. (2020), who expected a loss of 30% of the *A. microtis* distribution area by 2027, due to forest loss. Based on the last three years of high deforestation rates in the Amazon (PRODES, 2022), and using a pessimistic projection for the future, the loss of area of occurrence of this species could be much more severe.

Considering habitat selection, often, the choice of an animal can later affect its survival or reproductive success (Hutto, 1985; Kristan, 2003; Stamps & Swaisgood, 2007). In the case of altered environments in the Amazon, it is possible that the more significant attraction for open habitats by the species *C. thous* and *S. venaticus*, may harm the survival and reproduction aptitude of these species in the future, in a pessimistic context of degradation of the Amazon Forest. The preference of *C. thous* for anthropogenic environments was evident in our GLM analyses, where forest and natural open areas negatively influenced the occurrence of this species. *S. venaticus* is more positively influenced by natural open areas and negatively affected by open anthropized areas. However, more than 20% of the Brazilian Amazon Forest has already been transformed into anthropogenic areas without forests (PRODES‐Terrabrasilis/INPE, 2022). Evaluating the areas of the potential distribution of the two species in the Amazon, we observed that a large portion overlaps the already degraded area, especially in the so‐called “Arc of Deforestation.” These anthropized habitats at first probably can be as attractive as the preserved native habitats. This can happen when animals whose behaviors have been shaped by exposure to different environmental conditions in their evolutionary history are suddenly confronted with altered or new environments (Iwasa & Levin, 1995). However, the most significant loss of suitable area for the occurrence of *S. venaticus* in the future is precisely in these regions of current high anthropic pressure. In those situations, in which modified environments become attractive but which, in the medium and long term, may reduce the chances of survival of the species, we can consider it as an ecological trap (Gates & Gysel, 1978; Robertson & Hutto, 2006). *S. venaticus* is considered rare (de Moura Bubadué et al., 2015), and occurs in forest habitats and forest edges (Zuercher et al., 2005), open areas such as savanah (Zuercher, 2001), and altered habitats DeMatteo and Loiselle (2008). Despite not being a habitat specialist, *S. venaticus* is the most carnivorous canid in South America. This is due to the presence, in individuals of this species, of a large zygomatic arch, a short snout with a reduced number of molars and a pointed lower first molar (LM1), important adaptations for hypercarnivory, which increases the efficiency of meat consumption, consider as a specialist habitat (Beisiegel & Zuercher, 2005; de Moura Bubadué et al., 2015; Ewer, 1973; Van Valkenburgh & Koepfli, 1993).

Thresholds for the occurrence of *S. venaticus* appear to be less restrictive than forother species. DeMatteo and Loiselle (2008) mention that 20% of the potential distribution areas of *S. venaticus* were associated with degraded areas, using land‐use data from 1992 to 1993. However, Michalski (2010) recorded *S. venaticus* only in continuous regions of Forest, even applying an effort using camera traps in fragments adjacent to continuous areas. In our study, the current model showed broad distribution suitability in South America for this species, but with a significant loss in the future (the most extensive loss among the three species), especially in the Amazon region. The future loss of climatic niches in the Brazilian Amazon is concentrated both in forest areas and areas already anthropized, indicating that this species may suffer from the loss of high and low‐quality habitats. However, unlike *C. thou*, *S. venaticus* also uses forest habitats, which allows it to survive in areas that can be protected in the future in the Amazon.

Of the three species surveyed, *C. thous* appears to be the least demanding in terms of habitat, with an aptitude for open environments and tolerance for anthropogenic habitats (Ferraz et al., 2010). In the Amazon, this species is common in natural non‐forest environments, such as Canga Metalófila in Carajás, State of Pará (Carvalho et al., 2014), or in areas of the Amazon Cerrado in the State of Amapá (Coelho et al., 2014). In our occurrence database of the Amazon, we had no record of *C. thous* in areas of continuous forest and outside naturally open environments. On the other hand, *C. thous* has been commonly recorded in areas with eucalyptus (Coelho et al., 2014) and oil palm plantations (Mendes‐Oliveira et al., 2017), and may occur at the edge of adjacent forests, but not inside them. It also occurs in mining areas, with a high level of degradation and locations in the initial phase of forest recovery in the region of Paragominas State of Pará. Interestingly, two individuals of *C. thous* were recorded in 2020 in the city of Belém, State of Pará, in the Amazon (Mendes‐Oliveira, personal comm.). *Cerdocyon thous* does not seem to be distributed within forests, and deforestation has favored its expansion in the Amazon biome. The same occurred with *Chrysocyon brachyurus*, (maned wolf), which expanded its extent of occurrence in southwestern Amazonia (see Silva‐Diogo et al., 2020), precisely due to the conversion of forests into open areas in southeastern Amazonia (Silva‐Diogo et al., 2020).

Our current model showed more outstanding suitability of *C. thous* to more open environments, such as the Cerrado and more anthropized open areas in the Amazon, with restrictions to more forested areas, such as in the central and northwestern portions of the Amazon. GLM results also corroborate the conclusion that *C. thous* has been negatively influenced by forests. However, this species was also negatively influenced by natural open areas, suggesting a greater fitness for anthropogenic habitats over natural areas. Although canids generally do not show cursor adaptations like other terrestrial mammals (Smith & Savage, 1956), *C. thous* has a more cursorial form than the other two canid species studied in this research (Penrose, 2019). Longer limbs favor more extended travel, especially in open areas. Faced with a scenario of degradation and deforestation, this may be a morphological adaptation that favors the entry of this species into degraded, previously forested environments. Another characteristic that confers an advantage to the ecological plasticity of *C. thous*, about *A. microtis* and *S. venaticus* in the Amazon, is its greater tendency to omnivory. The ability of this species to have a poor or meatless diet for long periods, feeding opportunistically on what the environment offers (de Oliveira, 2009; Macdonald & Courtenay, 1996), allows it a more remarkable ability to thrive in a broader range of backgrounds and conditions. Considering all these characteristics, we can affirm that the deforestation and degradation of the Amazon Forest have favored the occurrence and dispersion of the *C. thous* species. However, in the long term, we cannot guarantee that this species is not being lured into an ecological trap. As already mentioned for *S. venaticus*, we observed a significant loss of suitable areas for *C. thous* in the future, especially in regions already open or currently degraded by anthropogenic action, mainly in the southern limits of the Amazon Forest and the northeast of this biome. These climate niche loss projections suggest that impoverished environments currently suitable for *C. thous* may not perpetuate in the future. Thus, these current anthropogenic environments may be working as an ecological trap for *C. thous* in the Amazon.

Evolutionarily seen, the climate has been a more decisive factor than the competition in explaining morphological variation and habitat options in canids. de Moura Bubadué et al. (2015) showed that the skull morphology of *A. microtis* and *S. venaticus* is more related to warm, humid, and less seasonal environments than *C. thous*. The use of different habitats and different diets reduces competition between them. In this way, the coexistence of these species can be compromised not by competition between them but, on the contrary, by differences in climatic adaptations and habitat options, as our results show.

Ecological traps occur when species erroneously choose niches where their suitability is lower than in others after changes in the environment (Robertson et al., 2013). Considering the evolutionary history of species, environments altered by human actions could provide false clues to their adaptation (Schlaepfer et al., 2002). In the medium and long term, these ecological traps can lead to a significant decrease or even extinction of populations in these habitats before adaptation to the new modified environment has occurred (Hale & Swearer, 2016). For *C. thous* and *S. venaticus*, we believe that rapid changes in landscapes (habitat degradation) will lead to “wrong choices” for environments that will soon have large climatic variations, leading these species to be trapped in these ecosystems, without time to adapt to them.

*Atelocynus microtis* appear to be forest‐dependent and must rely on the goodwill of decision‐makers to be maintained in the future. However, even though *C. thous* is least dependent on the Amazon Forest, this species may probably be affected in the future due to the ecological traps that the region can offer. *S. venaticus*, can also undergo the same process, but perhaps more drastically due to the lower ecological plasticity of this species compared to *C. thous*. Using the canid species as a model, gave us the opportunity to investigate these ecological effects that can affect a large part of the Amazonian fauna in the current scenario. The future perspectives for preserving terrestrial vertebrate fauna in this region are pretty pessimistic. When it comes to the conservation of impoverished regions in the Amazon, the theory of ecological traps must be studied and discussed at the same level that habitat loss is considered a decisive criterion for biodiversity threat.

### SIGNIFICANCE STATEMENT

Considering the high degree of environmental degradation and deforestation in the Amazon Forest, understanding how species will respond to anthropogenic threats is critical to conservation. We believe that the newspaper Ecology and Evolution has visibility to show the ecological threats that have been occurring in the Amazon Forest.

## AUTHOR CONTRIBUTIONS

**Geovana de Oliveira:** Data curation (lead); formal analysis (lead); methodology (lead); writing – original draft (lead); writing – review and editing (equal). **Arleu Barbosa Viana‐Junior:** Formal analysis (equal); methodology (supporting); writing – review and editing (supporting). **Paulo Henrique Santos Trindade:** Data curation (supporting); formal analysis (supporting); methodology (supporting); writing – original draft (supporting). **Iara Ramos dos dos Santos:** Data curation (supporting); formal analysis (supporting); funding acquisition (supporting); project administration (supporting); resources (supporting); writing – review and editing (equal). **Paula Cristina Rodrigues de Almeida‐Maués:** Data curation (equal); formal analysis (equal); investigation (supporting); methodology (equal); writing – review and editing (supporting). **Fernando Geraldo Carvalho:** Data curation (equal); formal analysis (equal); writing – original draft (supporting); writing – review and editing (supporting). **Daniel Paiva Silva:** Data curation (supporting); formal analysis (equal); investigation (supporting); methodology (supporting); writing – review and editing (supporting). **Øystein Wiig:** Conceptualization (supporting); formal analysis (supporting); funding acquisition (equal); investigation (supporting); supervision (supporting); writing – review and editing (equal). **Leonardo Sena:** Funding acquisition (equal); project administration (equal); supervision (equal); writing – review and editing (equal). **Ana Cristina Mendes‐Oliveira:** Conceptualization (lead); data curation (supporting); formal analysis (supporting); funding acquisition (lead); investigation (lead); methodology (supporting); project administration (lead); supervision (lead); writing – original draft (lead); writing – review and editing (lead).

## FUNDING INFORMATION

Part of the funding for this research was provided by the Biodiversity Research Consortium Brazil‐Norway (BRC).

## CONFLICT OF INTEREST STATEMENT

We declare that the authors of this article have no conflicts of interest.

## Supporting information


Table S1.

Table S2.

Table S3.

Table S4.

Table S5.

Table S6.

Figure S1.

Figure S2.

## Data Availability

We confirm that the entire database used in this article is available in the Appendix and supplementary material of this manuscript.
