# Supplementary figures and images for: Wild canids and the ecological traps facing the climate change and deforestation in the Amazon Forest
Source: Ecol Evol. 2023 Jun 9;13(6):e10150. doi: 10.1002/ece3.10150 (PMC10251424; doi:10.1002/ece3.10150)

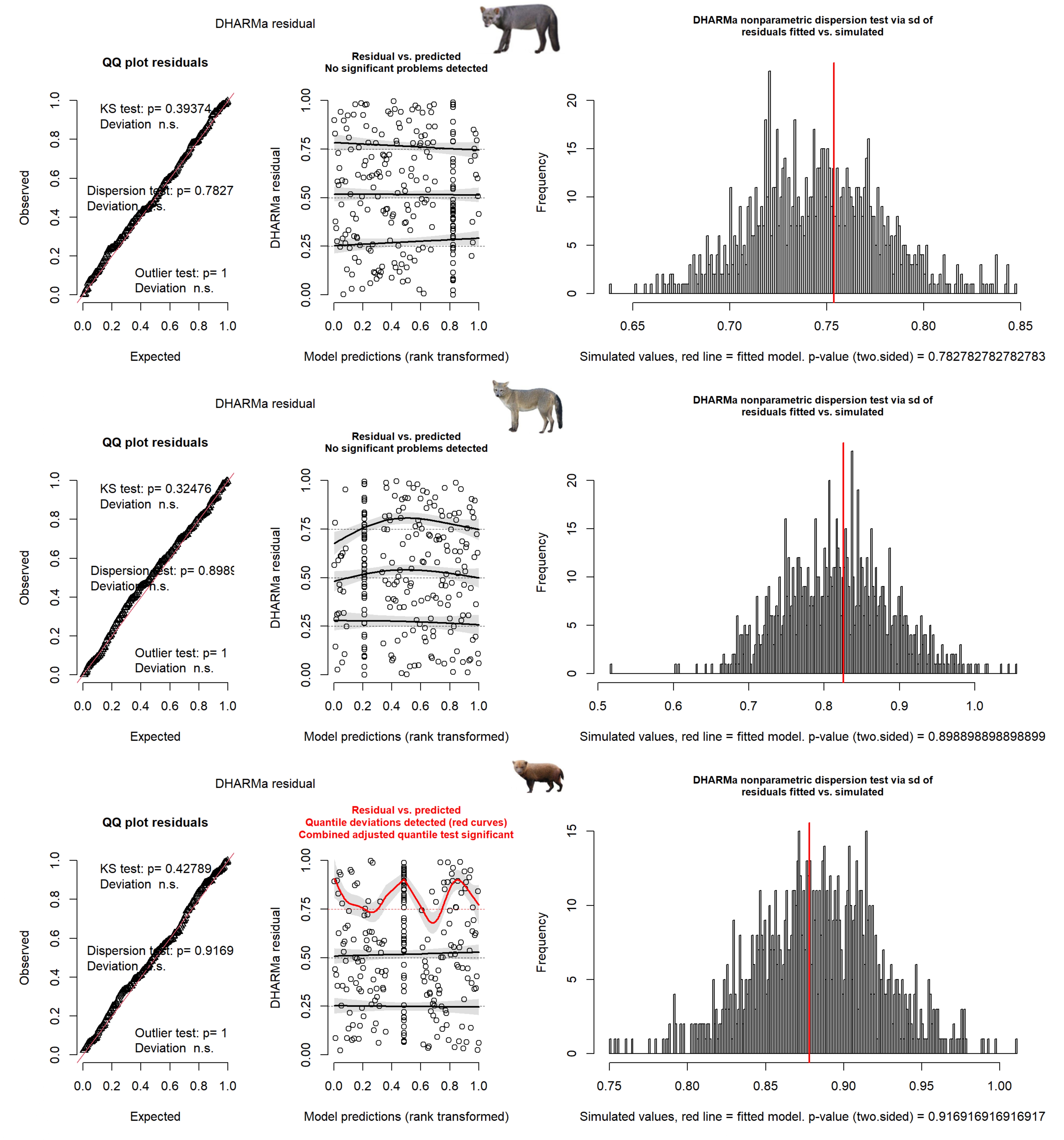

Supplement: Supplementary file 7 — Figure S1. [file ECE3-13-e10150-s001.jpg]

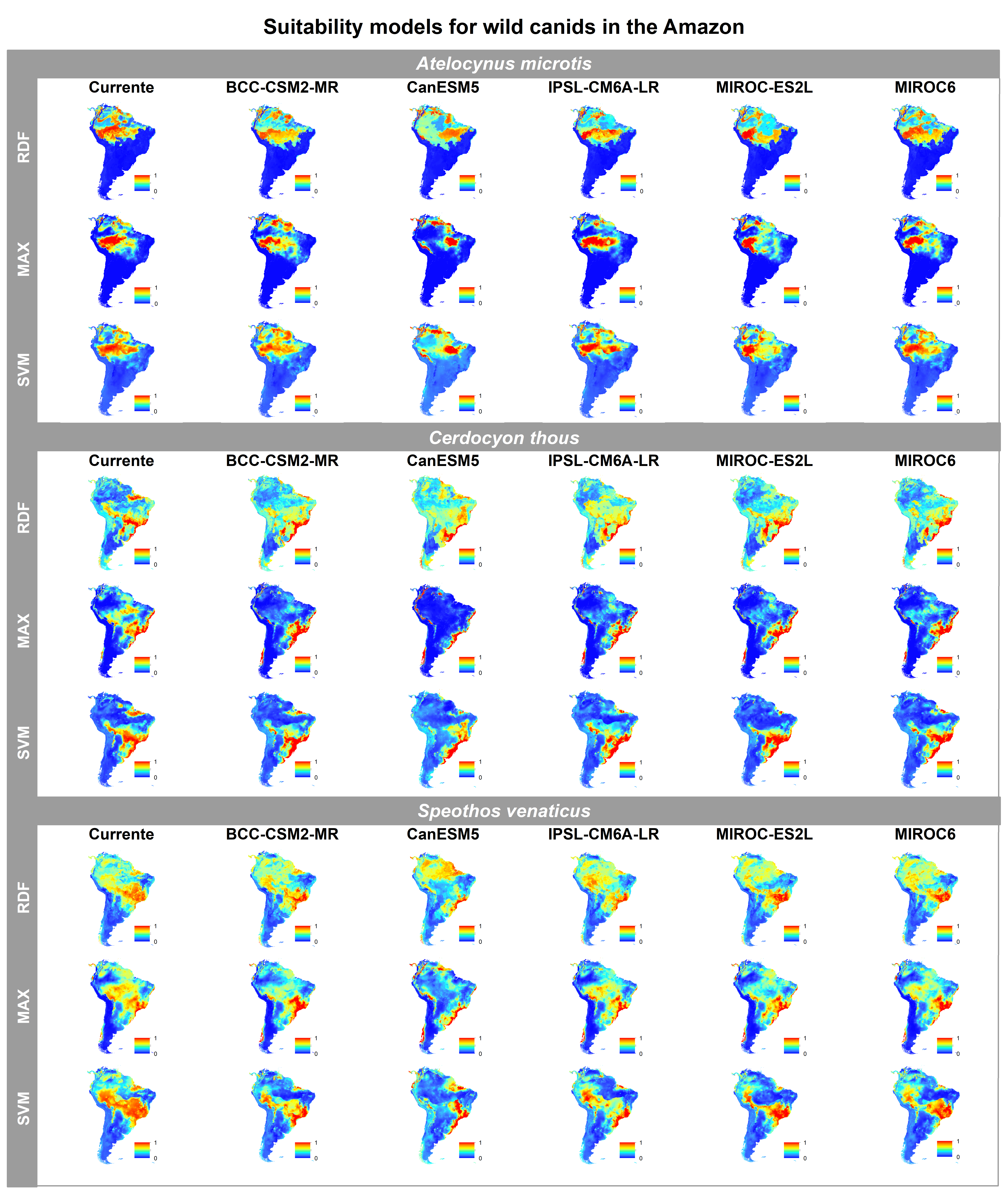

Supplement: Supplementary file 8 — Figure S2. [file ECE3-13-e10150-s007.png]
